# Supplementary material for: Pregnancy and neonatal outcomes in COVID-19: study protocol for a global registry of women with suspected or confirmed SARS-CoV-2 infection in pregnancy and their neonates, understanding natural history to guide treatment and prevention
Source: BMJ Open. 2021 Jan 29;11(1):e041247. doi: 10.1136/bmjopen-2020-041247 (PMC7849873; doi:10.1136/bmjopen-2020-041247)
Supplement: Supplementary data [file bmjopen-2020-041247supp001.pdf]

PAN-COVID

PREGNANCY AND NEONATAL OUTCOMES IN COVID-19

Imperial College London

Pregnancy and Neonatal Outcomes in COVID-19: A global registry of women affected by COVID-19 in pregnancy and their neonates, understanding natural history to guide treatment and prevention

Invitation to participate in early pregnancy

We are inviting women who have had suspected COVID-19 or confirmed SARS-CoV-2 infection (the virus that causes COVID-19) in their pregnancy to consent to join this research study, collecting information about their pregnancy. This form gives information about the study including the aims, risks and benefits of taking part.

WHAT YOU SHOULD KNOW ABOUT THIS RESEARCH STUDY:
What is the purpose of the study? This study aims to collect information about COVID-19 and SARS-CoV-2 in pregnancy from around the world into a register which the research team will use to share information with healthcare professionals around the world, allowing them to improve the care they give. We would like to find out more about the effect of COVID-19 on early pregnancy

Information will be held on a secure database and used anonymously to produce regular updates for healthcare professionals. The website with these reports and information is open to the public https://pan-covid.org/

Why have I been chosen? You have been chosen to consider taking part because you have had likely or confirmed COVID-19 during your pregnancy or just afterwards.

Do I have to take part? It is up to you to decide whether or not to take part. If you do decide to take part you will be given this information sheet to keep. If you decide to take part you are still free to withdraw at any time and without giving a reason. A decision to withdraw at any time, or a decision not to take part, will not affect the standard of care you receive. If you are happy to take part, we will ask for you to verbally give your consent, which will be recorded.

What will happen to me if I take part? Your healthcare professional will enter information about your pregnancy, the postnatal period and your baby into the secure study website. **This will include your NHS number, your date of birth, the key dates in the pregnancy and information about your health and the outcomes of the pregnancy. You will not be contacted again. We will use the NHS numbers to link the data collected to your routinely available health information**

What are the possible benefits of taking part? There will be no direct benefit to you from taking part in this study. We aim to use the information collected for this register to improve the understanding of COVID-19 in pregnancy and help healthcare professionals to improve treatment and prevention of the disease.

What are the possible risks of taking part? There are no risks that we can foresee from taking part in this study as we will only be collecting data.

What will happen to the results of the research study? Results of this study will be published on the study website, in medical journals and presented at medical conferences. You will not be identified in any report or publication.

Who is organising and funding the research? This study is funded by the UK Medical Research Council

Who has reviewed the study? This study was given a favourable ethical opinion for conduct in the NHS by Haydock Research Ethics Committee.

What if something goes wrong? If you are harmed by taking part in this research project, there are no special compensation arrangements. If you are harmed due to someone's negligence, then you may have grounds for a legal action. Regardless of this, if you wish to complain, or have any concerns about any aspect of the way you have been treated during the course of this study then you should immediately inform the Investigator Christoph Lees, c.lees@imperial.ac.uk . The normal National Health Service complaints mechanisms are also available to you

PAN-COVID PIS Early Pregnancy v1 19th May 2020 IRAS ID 2826505

CARDIFF UNIVERSITY PRIFYSGOL CAERDYDD

PAN-COVID

PREGNANCY AND NEONATAL OUTCOMES IN COVID-19

Imperial College London

Pregnancy and Neonatal Outcomes in COVID-19: A global registry of women affected by COVID-19 in pregnancy and their neonates, understanding natural history to guide treatment and prevention

How will we use information about you and your baby? Imperial College London is the sponsor for this study and will act as the data controller for this study. This means that we are responsible for looking after your information and using it properly. Imperial College London will keep your personal data for 10 years after the study has completed in relation to primary research data. We will need to use information from your medical records for this research project.

This information will include information about your pregnancy and general health. People will use this information to do the research or to check your records to make sure that the research is being done properly. We will keep all information about you safe and secure.

Legal basis As a university we use personally-identifiable information to conduct research to improve health, care and services. As a publicly-funded organisation, we have to ensure that it is in the public interest when we use personally-identifiable information from people who have agreed to take part in research. This means that when you agree to take part in a research study, we will use your data in the ways needed to conduct and analyse the research study. Health and care research should serve the public interest, which means that we have to demonstrate that our research serves the interests of society as a whole. We do this by following the UK Policy Framework for Health and Social Care Research.

International transfers There may be a requirement to transfer information to countries outside the European Economic Area (for example, to a research partner). Where this information contains your personal data, Imperial College London will ensure that it is transferred in accordance with data protection legislation. If the data is transferred to a country which is not subject to a European Commission (**EC**) adequacy decision in respect of its data protection standards, Imperial College London will enter into a data sharing agreement with the recipient organisation that incorporates EC approved standard contractual clauses that safeguard how your personal data is processed.

Sharing your information with others For the purposes referred to in this privacy notice and relying on the bases for processing as set out above, we will share your personal data with certain third parties.

- Other Imperial College employees, agents, contractors and service providers (for example, suppliers of printing and mailing services, email communication services or web services, or suppliers who help us carry out any of the activities described above). Our third party service providers are required to enter into data processing agreements with us. We only permit them to process your personal data for specified purposes and in accordance with our policies.
- Cardiff University, who are running the database for the study

What are your choices about how your information is used? You can stop being part of the study at any time, without giving a reason, by emailing c.lees@imperial.ac.uk but we will keep information about you that we already have.

- If you choose to stop taking part in the study, we would like to continue collecting information about your health from your hospital. If you do not want this to happen, tell us and we will stop.
- We need to manage your records in specific ways for the research to be reliable. This means that we won't be able to let you see or change the data we hold about you.

Where can you find out more about how your information is used You can find out more about how we use your information

- at www.hra.nhs.uk/information-about-patients/
- by asking one of the research team
- by sending an email to pan-covid@cardiff.ac.uk

Complaint If you wish to raise a complaint on how we have handled your personal data, please contact Imperial College London's Data Protection Officer via email at dpo@imperial.ac.uk, via telephone on 020 7594 3502 and/or via post at Imperial College London, Data Protection Officer, Faculty Building Level 4, London SW7 2AZ. If you are not satisfied with our response or believe we are processing your personal data in a way that is not lawful you can complain to the Information Commissioner's Office (ICO). The ICO does recommend that you seek to resolve matters with the data controller (us) first before involving the regulator. Once we have finished the study, we will keep some of the data so we can check the results. We will write our reports in a way that no-one can work out that you took part in the study.

PAN-COVID PIS Early Pregnancy v1 19th May 2020 IRAS ID 2826505

CARDIFF UNIVERSITY PRIFYSGOL CAERDYDD
