# Supplementary material for: Pregnancy and neonatal outcomes in COVID-19: study protocol for a global registry of women with suspected or confirmed SARS-CoV-2 infection in pregnancy and their neonates, understanding natural history to guide treatment and prevention
Source: BMJ Open. 2021 Jan 29;11(1):e041247. doi: 10.1136/bmjopen-2020-041247 (PMC7849873; doi:10.1136/bmjopen-2020-041247)
Supplement: Author's manuscript [file bmjopen-2020-041247.draft_revisions.pdf]

BMJ Open is committed to open peer review. As part of this commitment we make the peer review history of every article we publish publicly available.

When an article is published we post the peer reviewers' comments and the authors' responses online. We also post the versions of the paper that were used during peer review. These are the versions that the peer review comments apply to.

The versions of the paper that follow are the versions that were submitted during the peer review process. They are not the versions of record or the final published versions. They should not be cited or distributed as the published version of this manuscript.

BMJ Open is an open access journal and the full, final, typeset and author-corrected version of record of the manuscript is available on our site with no access controls, subscription charges or pay-per-view fees (http://bmjopen.bmj.com).

If you have any questions on BMJ Open's open peer review process please email info.bmjopen@bmj.com

BMJ Open

# Pregnancy and Neonatal Outcomes in COVID-19: Study protocol for a global registry of women with suspected or confirmed SARS-CoV-2 infection in pregnancy and their neonates, understanding natural history to guide treatment and prevention



| | |
|---|---|
| Journal: | *BMJ Open* |
| Manuscript ID | bmjopen-2020-041247 |
| Article Type: | Protocol |
| Date Submitted by the Author: | 03-Jun-2020 |





| | |
|---|---|
| Complete List of Authors: | Banerjee, Jayanta; Imperial College Healthcare NHS Trust, Department of Neonatology; Imperial College London Faculty of Medicine, Metabolism, Digestion and Reproduction<br>Mullins, E; Imperial College London, metabolism, digestion and reproduction; Imperial College<br>Townson, Julia; Cardiff University, Centre for Trials Research<br>Playle, Rebecca; Cardiff University, Centre for Trials Research<br>Shaw, Caroline; Imperial College London, Institute for reproductive and developmental biology<br>Kirby, Nigel; Cardiff University, Centre for Trials Research<br>Munnery, Kim; Cardiff University, Centre for Trials Research<br>Bourne, Tom; Imperial College London, Obstetrics and Gynaecology<br>Teoh, Tg; Imperial College Healthcare NHS Trust, Obstetrics and Gynaecology<br>Dhanjal, Mandish; Imperial College Healthcare NHS Trust, Obstetrics and Gynaecology<br>Poon, Liona; The Chinese University of Hong Kong, Obstetrics and Gynaecology<br>Wright, Alison; Royal Free London NHS Foundation Trust, Obstetrics and Gynaecology<br>Lees, Christoph; Imperial College London, Obstetrics and Gynaecology, Queen Charlotte's and Chelsea Hospital |





| | |
|---|---|
| Keywords: | PUBLIC HEALTH, OBSTETRICS, NEONATOLOGY, INFECTIOUS DISEASES |
| | |



SCHOLARONE™
Manuscripts

BMJ

*I, the Submitting Author has the right to grant and does grant on behalf of all authors of the Work (as defined in the below author licence), an exclusive licence and/or a non-exclusive licence for contributions from authors who are: i) UK Crown employees; ii) where BMJ has agreed a CC-BY licence shall apply, and/or iii) in accordance with the terms applicable for US Federal Government officers or employees acting as part of their official duties; on a worldwide, perpetual, irrevocable, royalty-free basis to BMJ Publishing Group Ltd ("BMJ") its licensees and where the relevant Journal is co-owned by BMJ to the co-owners of the Journal, to publish the Work in this journal and any other BMJ products and to exploit all rights, as set out in our licence.*

*The Submitting Author accepts and understands that any supply made under these terms is made by BMJ to the Submitting Author unless you are acting as an employee on behalf of your employer or a postgraduate student of an affiliated institution which is paying any applicable article publishing charge ("APC") for Open Access articles. Where the Submitting Author wishes to make the Work available on an Open Access basis (and intends to pay the relevant APC), the terms of reuse of such Open Access shall be governed by a Creative Commons licence – details of these licences and which Creative Commons licence will apply to this Work are set out in our licence referred to above.*

*Other than as permitted in any relevant BMJ Author's Self Archiving Policies, I confirm this Work has not been accepted for publication elsewhere, is not being considered for publication elsewhere and does not duplicate material already published. I confirm all authors consent to publication of this Work and authorise the granting of this licence.*

For peer review only

# Pregnancy and Neonatal Outcomes in COVID-19: Study protocol for a global registry of women with suspected or confirmed SARS-CoV-2 infection in pregnancy and their neonates, understanding natural history to guide treatment and prevention

Short title: Pregnancy and Neonatal outcomes in COVID-19 international registry

Author list

Joint first author
Dr Jayanta Banerjee, MD (Res)
Neonatal consultant and Hon Clinical Sen Lecturer
Du Cane Road, London, W12 0HS
Imperial College Healthcare NHS Trust
Institute of Reproductive and Developmental Biology, Imperial College London
jayanta.banerjee@ nhs.net
+44 77 7182 6045

Joint first author
Dr Edward Mullins, PhD
Clinical Lecturer in Obstetrics and Gynaecology
Du Cane Road, London, W12 0HS
Department of Metabolism, Digestion and Reproduction, Imperial College London
Edward.mullins@ imperial.ac.uk
+44 78 8102 9947

Julia Townson, BA
Research Fellow/Senior Trial Manager,
Centre for Trials Research, College of Biomedical and Life Sciences
Cardiff University
townson@ cardiff.ac.uk
+44 29 2068 7606

Dr Rebecca Playle, PhD
Deputy Director of Statistics
Centre for Trials Research, College of Biomedical and Life Sciences
Cardiff University
playlera@ cardiff.ac.uk
+44 29 2068 7516

Dr Caroline Shaw
Clinical Lecturer in Obstetrics
Du Cane Road, London, W12 0HS
Institute of Reproductive and Developmental Biology, Imperial College London
c.shaw13@ imperial.ac.uk

Nigel Kirby
Senior Data Manager
Centre for Trials Research, College of Biomedical and Life Sciences
Cardiff University

For peer review only

KirbyN@cardiff.ac.uk

Kim Munnery
Data Manager
Centre for Trials Research, College of Biomedical and Life Sciences
Cardiff University
MunneryK1@cardiff.ac.uk

Prof Tom Bourne, PhD
Professor of Practice, Imperial College London
Consultant Gyanecologist, Queen Charlotte's and Chelsea Hospital
tbourne@imperial.ac.uk
+44 77 6807 6797

Professor Tg Teoh,
Professor and Consultant Obstetrician
St Marys Hospital
Imperial College Healthcare NHS Trust
Tg.teoh@ nhs.net

Dr Mandish Dhanjal, BSc
Consultant Obstetrician and Gynaecologist
Queen Charlotte's and Chelsea Hospital
Imperial College Healthcare NHS Trust
Mandish.Dhanjal@ nhs.net
+44 7958 323066

Prof Liona C. Poon
Professor Department of Obstetrics and Gynaecology
The Chinese University of Hong Kong
liona.poon@ cuhk.edu.hk
+852 61089496

Dr Alison Wright
Consultant Obstetrician and Gynaecologist
Royal Free Hospital
Alison.Wright15@ nhs.net
+44 7702269002

Corresponding author
Prof Christoph Lees, MD
Professor of Obstetrics and Hon Consultant Obstetrician
Lead, Centre for Fetal Care
Imperial College Healthcare NHS Trust
Institute of Reproductive and Developmental Biology, Imperial College London
c.lees@imperial.ac.uk
+44 77 8526 8351

For peer review only

# Abstract

## Introduction

Previous novel coronavirus pandemics, Severe Acute Respiratory Syndrome (SARS) and Middle East Respiratory Syndrome (MERS), observed an association of infection in pregnancy with pre-term delivery, stillbirth and increased maternal mortality. Coronavirus disease 2019 (COVID-19), caused by SARS-CoV-2 infection, is the largest pandemic in living memory.

Rapid accrual of robust case data on women in pregnancy and their babies affected by suspected COVID-19 or confirmed SARS-CoV-2 infection will inform clinical management and preventative strategies in the current pandemic and future outbreaks.

## Methods and analysis

The Pregnancy And Neonatal outcomes in COVID-19 (PAN-COVID) registry is an observational study collecting focussed data on outcomes of pregnant mothers who have had suspected COVID-19 in pregnancy or confirmed SARS-CoV-2 infection and their neonates via a web-portal. Amongst the women recruited to the PAN-COVID registry, the study will evaluate the incidence of:

1. Miscarriage and pregnancy loss
2. FGR and stillbirth
3. Pre-term delivery
4. Vertical transmission (suspected or confirmed) and early-onset neonatal SARS-CoV-2 infection

Data will be centre based and collected on individual women and their babies. Verbal consent will be obtained, to reduce face-to-face contact in the pandemic whilst allowing identifiable data collection for linkage. Statistical analysis of the data will be carried out on a pseudonymised dataset by the study statistician. Regular reports will be distributed to collaborators on the study research questions.

## Ethics and dissemination

This study has received research ethics approval in the UK. For international centres, evidence of appropriate local approval will be required to participate, prior to entry of data to the database. The reports will be published regularly. The outputs of the study will be regularly disseminated to


For peer review only

participants and collaborators on the study website (https://pan-covid.org) and social media channels as well as dissemination to scientific meetings and journals.

## Study Registration number

The study is registered with ISRCTN68026880

For peer review only



For peer review only - http://bmjopen.bmj.com/site/about/guidelines.xhtml

Article summary

## Strengths and limitations of the study

- The PAN-COVID registry aims to collect an international dataset, which will enable us to answer a focussed set of questions related to pregnancy, maternal and neonatal outcomes of SARS-CoV-2 infection in pregnancy.

- There is a high likelihood of an adequate sample size to allow us to measure all the stated focussed outcomes of SARS-CoV-2 infection in pregnancy.

- The PAN-COVID protocol facilitates data sharing and collaboration with multiple global partners.

- This registry establishes a collaborative framework, which can be used to respond effectively to guide care for women in pregnancy and their neonates in future pandemics.

- Registry studies are generally unlikely to fully capture all cases and therefore carry risk of underestimating infection rates and over-estimating infection complication and fatality rates.

For peer review only

# Introduction

The majority of all new, emerging, or re-emerging viruses affecting the human population in the recent years are zoonoses, including HIV/AIDS, severe acute respiratory syndrome (SARS), the H5N1 strain of avian influenza, the 2009 pandemic H1N1 influenza virus, the Middle East respiratory syndrome (MERS), the Zika and Nipa viruses[1][2] and most recently Coronavirus disease 2019 (COVID-19) caused by SARS-CoV-2 virus. The spread of these infections in an immune-naïve human population, as shown by the recent COVID-19 pandemic poses serious strain on public health. To tackle such large pandemics, international collaborative and multidisciplinary approaches are necessary.

These should aim to identify those at highest risk of exposure to emerging pathogens, to characterise the culture and practices that modify their risk (e.g. the presence of live animal markets and the use of face masks in South-East Asia) and to develop preventative interventions accordingly [3][4]. Pregnant women infected with SARS or MERS were at increased risk of mortality and morbidities such as stillbirth, preterm birth and fetal growth restriction (FGR)[5][6]. Case series for pregnant women infected during outbreaks of SARS [7] or MERS[8] report a variety of different outcomes, with variable recording of diagnostic testing, maternal, fetal and neonatal outcomes, and the presence or absence of vertical transmission. Clinical outcomes appear worse for pregnant compared with non-pregnant women infected with SARS and H1N1 influenza[7][9].

As of the 12$^{th}$ May 2020, after disambiguation and removal of duplicate reporting, the rolling review of all cases worldwide conducted by Thornton et al.[10] contains 92 publications including details of 806 pregnant women and 674 fetuses. At the time of writing this protocol, 457 of the included women had delivered and were alive and 21 women had died. These women had delivered 428 babies, of which 94 were born pre-term, 17 were infected with COVID-19, and there were 15 neonatal deaths. The largest study to date has been from the UK (Knight et al) who reported 427 cases (247 competed pregnancies): there were 5 (1%) maternal deaths, 3 (1%) stillbirths, and 2 (1%) neonatal deaths[11]. There are currently limited data on the effect of COVID-19 on second trimester miscarriage [12] and no available data on first trimester miscarriage or growth restriction. The risk of reported probable vertical transmission of SARS-CoV-2 infection is estimated at 2.5% in women admitted to hospital with COVID-19; with case reports describing increased SARS-CoV-2 IgM levels in neonates, and pneumonia and



For peer review only - http://bmjopen.bmj.com/site/about/guidelines.xhtml

lymphopaenia in neonates with negative COVID-19 RT-PCR testing, principally from naso-pharyngeal swabs [13][14].

There are major knowledge gaps on the effect of COVID-19 on various stages of pregnancy, and its effect on fetuses in terms of growth restriction, prematurity and short and long term morbidities[15]. There is an urgent need to collect case data rapidly, to pool global data on the natural history of women affected by suspected COVID-19 or confirmed SARS-CoV-2 in pregnancy to inform treatment and implement preventative strategies in the current and future outbreaks. Published case series are almost always out of date when published and cases may overlap. A centre-based registry, gathering case data prospectively on the effect of SARS-CoV-2 infection from healthcare systems around the world offers a method to accrue clinical outcomes on key research questions from a variety of populations and healthcare systems without these limitations.

PAN-COVID registry will focus on miscarriage, fetal growth restriction, stillbirth, pre-term delivery and vertical transmission (suspected or confirmed) and early-onset neonatal SARS-CoV-2 infection and will include fields on ultrasound diagnosis and neonatal care not included in other more general studies. This study will identify cases which should also be reported to International Network of Obstetric Survey Systems (INOSS) population-based surveillance systems of confirmed COVID-19 cases admitted to hospital through existing reporting mechanisms. The UK's obstetric surveillance system (UKOSS) is a population surveillance study, reporting women in pregnancy hospitalized with COVID-19 or SARS-CoV-2 infection. The PAN-COVID registry is distinct in that it will collect, via a web portal, data on SARS-CoV-2 infections in pregnancy, whether the diagnosis is presumptive based on symptoms or following a positive test and whether the patient is admitted to hospital or not. Given that the SARS-CoV-2 virus is likely to affect a high proportion of the global population and adverse outcomes may influence policy and practice in a short time-frame, 'real-time' high level reporting on a regular basis will be valuable for UK and international clinicians and policy makers.

PAN-COVID registry will work collaboratively with approved studies and registries to ensure that the global impact of the pandemic is captured as fully as possible, with data sharing and linkage. PAN-COVID has established links with the UK National Neonatal Research Database (NNRD) (REC Reference: 16/LO/1093) who will provide data on neonatal outcomes, and the British Paediatric Surveillance Unit (BPSU) with whom there is in principle an agreement for data sharing.



For peer review only - http://bmjopen.bmj.com/site/about/guidelines.xhtml

In the UK, the PAN-COVID registry will signpost to UKOSS where the case definition requires a more detailed data collection. We are in discussions with several international studies to agree on a common dataset to enable future merging of information.

## Methods and analysis

This is an observational global pregnancy and neonatal register (PAN-COVID registry) collecting outcome data from women in pregnancy who have confirmed SARS-CoV-2 infection or signs and symptoms of COVID-19 during their pregnancy. The study is sponsored by Imperial College London and funded by UK Research Institute (UKRI) and NIHR.

The main study objectives are, firstly, to establish an UK and international disease registry for women with suspected COVID-19 or confirmed SARS-CoV-2 infection in pregnancy; in the UK this will be linked to neonatal data (from NNRD). Secondly, PAN-COVID investigators aim to publish regular reports focused on our principle research question: in women recruited to the PAN-COVID registry with 1) Suspected COVID-19 or 2) Confirmed SARS-CoV-2 infection, what is the incidence of (a) miscarriage, (b) fetal growth restriction and stillbirth, (c) pre-term birth and (d) Suspected or confirmed vertical transmission to the neonate and perinatal infection.

### Data entry

*Demographics* – date of birth, NHS number (or international equivalent), smoking status, last menstrual period, expected date of delivery, body mass index (BMI), history of previous pregnancies/miscarriages/loss/small for gestational age/delivery (pre-term/term), co-morbidities, medications, ethnicity, number of fetuses, structural abnormality on ultrasound.

*COVID-19 signs and symptoms* – fever, new persistent cough, anosmia, myalgia, diarrhoea, shortness of breath, fatigue, abdominal pain, chest pain, hoarseness of voice, loss of appetite, delirium.

*SARS-CoV-2 infection* – SARS CoV 2 test performed, date of test(s), results of test(s) (if available)

*COVID-19 treatment* – requirement for (including duration of) inpatient, level 2 or 3 critical care, supplementary oxygen, non-invasive ventilation, endo-tracheal intubation, extracorporeal membrane oxygenation, antiviral/other specific anti-COVID-19 therapy, iatrogenic delivery or termination due to maternal compromised from COVID-19.

*Maternal outcomes* – non-invasive ventilation, intubation and ventilation and maternal death.



For peer review only - http://bmjopen.bmj.com/site/about/guidelines.xhtml

*Co-morbidities* – Hypertension, respiratory disease, cardiovascular disease, renal disease, autoimmune disease, medications including aspirin, progesterone, others.

*Delivery details* – iatrogenic/spontaneous, date of delivery/miscarriage, mode of delivery, livebirth/stillbirth/miscarriage, birthweight, sex.

*Postnatal outcomes* – breastfeeding, baby cared for separately from mother until discharge

*Neonatal outcomes* – date of birth, birthweight, gestation at birth, APGAR at 5 minutes, neonatal death, COVID-19 transmission (neonate with positive swab/serology), congenital abnormality/deformation, date of discharge from hospital, re-admission up to 28 days of life.

## Study population

Study Centres will be asked to identify eligible women of childbearing age (18-50 years) at the time of an early pregnancy attendance or at delivery who have had suspected COVID-19 or confirmed SARS-CoV-2 infection in pregnancy. Case data will be entered into the online registry. The study will recruit women affected by suspected COVID-19 or confirmed SARS-CoV-2 infection from January 2020 and March 2021. Participants will be able to withdraw from the register at any time without affecting their medical care. Participants should inform any member of the study team of their wish to withdraw consent. When a participant withdraws consent, they should be asked whether they are willing to allow ongoing data collection, such as maternal and fetal outcomes, from their medical records. If the participant withdraws consent and does not wish to have any further data collected, then this will be recorded on the database and no further data entry will be possible.

## Outcomes

Assessment of outcomes will require follow up by individual healthcare professionals, by accessing medical records routinely available to them as part of the clinical care team. When a pregnant woman with SARs-CoV-2 infection or suspected COVID 19 is registered on the database, they will automatically be assigned a unique participant identification number. Date of birth and NHS number, or equivalent in non-UK centres, will be stored on the database. The limit for data collection will be 28 days after the delivery or pregnancy loss of the last woman registered. Linkage will allow data up to 2 years of age to be collected for pre-term babies in the UK with the NNRD database.

## Data collection pathway

For peer review only


For peer review only - http://bmjopen.bmj.com/site/about/guidelines.xhtml

The Centre for Trials Research (CTR), Cardiff University, has designed a registry and web portal for data entry for healthcare professionals. The CTR hosts and maintans the study web page (https://pan-covid.org) which allows maternity healthcare providers to register interest in submitting data to an online database. The data manager will manage the registration of healthcare professionals, keeping personal identifiable information on a separate, password protected, spreadsheet with restricted access. The study co-ordinator will manage registration of healthcare professionals and work with the data manager to provide login permission to those who wish to contribute to the register. Login permission will only be granted to healthcare professionals who have confirmed their email address, which must be from a bona fide healthcare or educational organisation and local agreement and ethical approval is in place.

Where a web portal cannot be accessed by the investigators, a bespoke spreadsheet has been designed to allow automatic uploading of data to the main registry database.

## Analysis

Statistical analysis of the data will be carried out a pseudonymised dataset containing NHS number (or equivalent international data field). Numerical data for cases reported and outcomes (miscarriage, FGR, stillbirth, pre-term delivery and vertical transmission) will be reported in anonymised, aggregate regular reports, published on the study website.

## Sample size

Pre-specified sample size estimation was not carried out given the aim of this observational study was to collate all consecutive eligible case outcomes in participating centres between data collection start and 18 months from start of data collection. However, it may be useful to provide an approximate guide as to the power that can be achieved for one sample, two sided differences of proportions with alpha at 5% and a reference proportion using historical data from the UK. These historical data may not hold during the current pandemic and may alter due to multiple reasons such as access to health care or behaviour changes. Contemporaneous data collected using other current national registries will provide true background rates for comparative purposes at the end of the study period. Generalising these data internationally may not be possible unless similar contemporaneous data exist for comparison.



For peer review only - http://bmjopen.bmj.com/site/about/guidelines.xhtml

The estimated representative pre-COVID pandemic incidences of miscarriage, SGA and stillbirth in the UK were 30%, 10%, 0.2% respectively. The expected outcome proportions during the COVID pandemic are 40%, 15% and 0.4% respectively. A sample size of 500 would allow the estimation of the width of 95% confidence intervals for the proportion of miscarriage as 40% +/- 4.2%, for SGA 15% +/- 2.9% and for still birth 0.4% +/-0.3%.

These figures are meant only as a guide and reference proportions may change over this period. Since attaining sufficient power for stillbirth requires by far the largest sample size then study recruitment targets could be based on this rarest of outcomes. This would then provide sufficient power to assess associations in other more common outcomes. These figures are based on comparisons within the UK only. Reference proportions vary by country but are of a similar order to those in the UK, hence all within-country comparisons would require equivalent sample sizes.

Appropriate quantitative analyses will be conducted by a study statistician. Proportional data for the study outcomes will be presented with 95% confidence intervals, by country, where numbers allow. Data and all appropriate documentation will be stored for a minimum of 10 years after the completion of the study, including the follow-up period.

## Ethics and dissemination

The Study Coordination Centre has obtained approval from the Haydock Research Ethics Committee (REC) and Health Regulator Authority (HRA), REC reference: 20/NW/0212 and had been considered to be an Urgent Public Health (UPH) study by the UK Clinical Research Network (UK CRN).

The study must also receive confirmation of capacity and capability from each participating NHS Trusts in the UK before accepting participants into the study or any research activity is carried out. The study will be conducted in accordance with the recommendations for physicians involved in research on human subjects adopted by the 18th World Medical Assembly, Helsinki 1964 and later revisions. The international participating centres will be required to obtain appropriate local approval from their centres. All those taking consent will be qualified and trained to take consent in a medical context. To reduce face to face consultations during the pandemic verbal consent will be obtained after provision of the participant information sheet: PIS (Appendix 1) which will have



For peer review only - http://bmjopen.bmj.com/site/about/guidelines.xhtml

details of how to contact the PAN-COVID study team, if they would like further information or to withdraw from the study and this will be recorded in the patients notes and on the study database. Where women have lost capacity to provide consent and have confirmed COVID-19, women will be assumed to provide consent. If they regained capacity, the study will be explained to them and they will be provided PIS (v.5.1 19/5/20), which will have details of how to contact the PAN-COVID study team, if they would like further information or to withdraw from the study. If they choose to withdraw, data pertaining to their care would be removed from the registry. Where feasible, the woman's partner or a family member will be provided with the PIS and asked if they feel able to advise on the presumed wishes and feelings of participants unable to consent for themselves and on their inclusion in the research. Their response will be recorded on the database and in the participants clinical notes. These models will give a reasonable opportunity for women to provide their data to the study, whilst being mindful not to over burden healthcare resources. Research has shown that within a pandemic, potential participants and their families are broadly agreeable to alternative methods of gaining consent, therefore we decided upon this verbal model of consent, as a pragmatic solution to ensure women are informed of how their data will be used, and alleviate additional burden to healthcare resources [16]. The Chief Investigators (CCL and EM) will preserve the confidentiality of participants taking part in the study and is registered under the General Data Protection Regulation (GDPR) 2018. In terms of indemnity, Imperial College London holds negligent harm and non-negligent harm insurance policies which apply to this study. Imperial College London will act as the Sponsor for this study. Delegated responsibilities will be assigned to the NHS trusts taking part in this study. The study may be subject to inspection and audit by Imperial College London under their remit as sponsor and other regulatory bodies to ensure adherence to GCP and the UK Policy Framework for Health and Social Care Research.

## Data security

Cardiff University has strict policies and processes in place to ensure adherence to the General Data Protection Regulation 2018. The University is registered on the Information Commissioner's Office Data Protection register, number Z6549747. All data collected on the Registry will be held on Cardiff University servers, which have a high level of security. CTR database developers have run a security check on the trials.cardiff.ac.uk server and it has passed to an acceptable standard to EU and non-EU centres.



For peer review only - http://bmjopen.bmj.com/site/about/guidelines.xhtml

The person allocating Centre IDs and logins will not have access to the database fields containing personal information. For healthcare professionals to enter follow up data they will need to maintain a secure spreadsheet (password protected) with participants' identifiable information logged against their unique participant identification number.

To reduce any potential risk of identification of study participants, access will be restricted to the section of the database containing identifiable information (NHS number, date of birth, ethnicity) to those responsible for maintaining the database (two individuals). This will mean that the CTR members of the research team (two different individuals) responsible for allocating the unique centre and participant IDs will not be able access any identifiable data. In addition, all other CTR members of the research team will be blinded to which centre ID relates to which centre. For statistical analyses, a dataset will be prepared by the database developers which will have all NHS numbers, and the international equivalent, removed, reducing possible identification of participants further.

## Dissemination

Regular reports of case data will be made available on the study website. Although members of the public were not involved in the study design as it is broadly a registry, the maternity and neonatal focus groups will be involved in the dissemination of the outputs. The outputs of the PAN-COVID registry will be presented in national and international conferences, lay and digital social media and published in peer-reviewed scientific journals which will be made available as open-access, as per the NIHR's publication policy.

For peer review only

# References

[1] D. Di Mascio *et al.*, "Outcome of Coronavirus spectrum infections (SARS, MERS, COVID 1 -19) during pregnancy: a systematic review and meta-analysis," *Am. J. Obstet. Gynecol. MFM*, 2020.

[2] M. Elisabeth Lopes Moreira *et al.*, "Neurodevelopment in infants exposed to Zika virus in utero," *New England Journal of Medicine*. 2018.

[3] K. J. Olival, P. R. Hosseini, C. Zambrana-Torrelio, N. Ross, T. L. Bogich, and P. Daszak, "Host and viral traits predict zoonotic spillover from mammals," *Nature*, 2017.

[4] D. Joly *et al.*, "The first phase of PREDICT: Surveillance for emerging infectious zoonotic diseases of wildlife origin (2009-2014)," *Int. J. Infect. Dis.*, 2016.

[5] A. M. Li and P. C. Ng, "Severe acute respiratory syndrome (SARS) in neonates and children," *Archives of Disease in Childhood: Fetal and Neonatal Edition*. 2005.

[6] C. C. Shek *et al.*, "Infants born to mothers with severe acute respiratory syndrome.," *Pediatrics*, 2003.

[7] S. F. Wong *et al.*, "Pregnancy and perinatal outcomes of women with severe acute respiratory syndrome," *Am. J. Obstet. Gynecol.*, 2004.

[8] S. H. Alfaraj, J. A. Al-Tawfiq, and Z. A. Memish, "Middle East Respiratory Syndrome Coronavirus (MERS-CoV) infection during pregnancy: Report of two cases & review of the literature," *Journal of Microbiology, Immunology and Infection*. 2019.

[9] M. Pierce, J. J. Kurinczuk, P. Spark, P. Brocklehurst, and M. Knight, "Perinatal outcomes after maternal 2009/H1N1 infection: National cohort study," *BMJ*, 2011.

[10] J. et al Thornton, "Ripe tomato.org." [Online]. Available: https://ripe-tomato.org/2020/03/31/covid-19-in-pregnancy-running-totals/.

[11] M. Knight et al., "Characteristics and outcomes of pregnant women hospitalised with confirmed SARS-CoV-2 infection in the UK: a national cohort study using the UK Obstetric Surveillance System (UKOSS) The UK Obstetric Surveillance System SARS-CoV-2 Infection in Pregnancy Co," 2020.

[12] D. Baud *et al.*, "Second-Trimester Miscarriage in a Pregnant Woman With SARS-CoV-2 Infection," *JAMA - J. Am. Med. Assoc.*, 2020.

[13] H. Zeng *et al.*, "Antibodies in Infants Born to Mothers with COVID-19 Pneumonia," *JAMA - Journal of the American Medical Association*. 2020.

For peer review only

[14] C. Fan *et al.*, “Perinatal Transmission of COVID-19 Associated SARS-CoV-2: Should We Worry?,” *Clin. Infect. Dis.*, 2020.

[15] H. Liu, L.-L. Wang, S.-J. Zhao, J. Kwak-Kim, G. Mor, and A.-H. Liao, “Why are pregnant women susceptible to viral infection: an immunological viewpoint?,” *J. Reprod. Immunol.*, 2020.

[16] N. H. Gobat *et al.*, “Key stakeholder perceptions about consent to participate in acute illness research: A rapid, systematic review to inform epi/pandemic research preparedness,” *Trials*, 2015.

For peer review only

# Appendix

Participant information leaflet v.5.1 19/5/20

# Authors' contributions

CL and EM conceived of the study and prepared the study design. JB, TB and JT helped in preparing the study design. JB prepared the first draft. All authors contributed to the manuscript and agreed the final draft.

# Funding statement

The PAN-COVID registry is funded by the United Kingdom Research Institute (UKRI) and National Institute of Health and Research (NIHR) through COVID-19 Rapid Response Call 2, grant reference MC_PC 19066

# Competing interest statement

None declared

For peer review only



For peer review only - http://bmjopen.bmj.com/site/about/guidelines.xhtml

PAN-COVID

# PREGNANCY AND NEONATAL OUTCOMES IN COVID-19

Imperial College London

Pregnancy and Neonatal Outcomes in COVID-19: A global registry of women affected by COVID-19 in pregnancy and their neonates, understanding natural history to guide treatment and prevention

## Invitation to participate in early pregnancy

We are inviting women who have had suspected COVID-19 or confirmed SARS-CoV-2 infection (the virus that causes COVID-19) in their pregnancy to consent to join this research study, collecting information about their pregnancy. This form gives information about the study including the aims, risks and benefits of taking part.

**WHAT YOU SHOULD KNOW ABOUT THIS RESEARCH STUDY:**

**What is the purpose of the study?** This study aims to collect information about COVID-19 and SARS-CoV-2 in pregnancy from around the world into a register which the research team will use to share information with healthcare professionals around the world, allowing them to improve the care they give. We would like to find out more about the effect of COVID-19 on early pregnancy

Information will be held on a secure database and used anonymously to produce regular updates for healthcare professionals. The website with these reports and information is open to the public https://pan-covid.org/

**Why have I been chosen?** You have been chosen to consider taking part because you have had likely or confirmed COVID-19 during your pregnancy or just afterwards.

**Do I have to take part?** It is up to you to decide whether or not to take part. If you do decide to take part you will be given this information sheet to keep. If you decide to take part you are still free to withdraw at any time and without giving a reason. A decision to withdraw at any time, or a decision not to take part, will not affect the standard of care you receive. If you are happy to take part, we will ask for you to verbally give your consent, which will be recorded.

**What will happen to me if I take part?** Your healthcare professional will enter information about your pregnancy, the postnatal period and your baby into the secure study website. **This will include your NHS number, your date of birth, the key dates in the pregnancy and information about your health and the outcomes of the pregnancy. You will not be contacted again. We will use the NHS numbers to link the data collected to your routinely available health information**

**What are the possible benefits of taking part?** There will be no direct benefit to you from taking part in this study. We aim to use the information collected for this register to improve the understanding of COVID-19 in pregnancy and help healthcare professionals to improve treatment and prevention of the disease.

**What are the possible risks of taking part?** There are no risks that we can foresee from taking part in this study as we will only be collecting data.

**What will happen to the results of the research study?** Results of this study will be published on the study website, in medical journals and presented at medical conferences. You will not be identified in any report or publication.

**Who is organising and funding the research?** This study is funded by the UK Medical Research Council

**Who has reviewed the study?** This study was given a favourable ethical opinion for conduct in the NHS by Haydock Research Ethics Committee.

**What if something goes wrong?** If you are harmed by taking part in this research project, there are no special compensation arrangements. If you are harmed due to someone's negligence, then you may have grounds for a legal action. Regardless of this, if you wish to complain, or have any concerns about any aspect of the way you have been treated during the course of this study then you should immediately inform the Investigator Christoph Lees, c.lees@imperial.ac.uk . The normal National Health Service complaints mechanisms are also available to you

CARDIFF UNIVERSITY
PRIFYSGOL CAERDYDD


For peer review only - http://bmjopen.bmj.com/site/about/guidelines.xhtml

PAN-COVID

# PREGNANCY AND NEONATAL OUTCOMES IN COVID-19

Imperial College London

Pregnancy and Neonatal Outcomes in COVID-19: A global registry of women affected by COVID-19 in pregnancy and their neonates, understanding natural history to guide treatment and prevention

**How will we use information about you and your baby?** Imperial College London is the sponsor for this study and will act as the data controller for this study. This means that we are responsible for looking after your information and using it properly. Imperial College London will keep your personal data for 10 years after the study has completed in relation to primary research data. We will need to use information from your medical records for this research project.

This information will include information about your pregnancy and general health. People will use this information to do the research or to check your records to make sure that the research is being done properly. We will keep all information about you safe and secure.

**Legal basis** As a university we use personally-identifiable information to conduct research to improve health, care and services. As a publicly-funded organisation, we have to ensure that it is in the public interest when we use personally-identifiable information from people who have agreed to take part in research. This means that when you agree to take part in a research study, we will use your data in the ways needed to conduct and analyse the research study. Health and care research should serve the public interest, which means that we have to demonstrate that our research serves the interests of society as a whole. We do this by following the UK Policy Framework for Health and Social Care Research.

**International transfers** There may be a requirement to transfer information to countries outside the European Economic Area (for example, to a research partner). Where this information contains your personal data, Imperial College London will ensure that it is transferred in accordance with data protection legislation. If the data is transferred to a country which is not subject to a European Commission (**EC**) adequacy decision in respect of its data protection standards, Imperial College London will enter into a data sharing agreement with the recipient organisation that incorporates EC approved standard contractual clauses that safeguard how your personal data is processed.

**Sharing your information with others** For the purposes referred to in this privacy notice and relying on the bases for processing as set out above, we will share your personal data with certain third parties.

- Other Imperial College employees, agents, contractors and service providers (for example, suppliers of printing and mailing services, email communication services or web services, or suppliers who help us carry out any of the activities described above). Our third party service providers are required to enter into data processing agreements with us. We only permit them to process your personal data for specified purposes and in accordance with our policies.
- Cardiff University, who are running the database for the study

**What are your choices about how your information is used?** You can stop being part of the study at any time, without giving a reason, by emailing c.lees@imperial.ac.uk but we will keep information about you that we already have.

- If you choose to stop taking part in the study, we would like to continue collecting information about your health from your hospital. If you do not want this to happen, tell us and we will stop.
- We need to manage your records in specific ways for the research to be reliable. This means that we won't be able to let you see or change the data we hold about you.

**Where can you find out more about how your information is used** You can find out more about how we use your information

- at www.hra.nhs.uk/information-about-patients/
- by asking one of the research team
- by sending an email to pan-covid@cardiff.ac.uk

**Complaint** If you wish to raise a complaint on how we have handled your personal data, please contact Imperial College London's Data Protection Officer via email at dpo@imperial.ac.uk, via telephone on 020 7594 3502 and/or via post at Imperial College London, Data Protection Officer, Faculty Building Level 4, London SW7 2AZ. If you are not satisfied with our response or believe we are processing your personal data in a way that is not lawful you can complain to the Information Commissioner's Office (ICO). The ICO does recommend that you seek to resolve matters with the data controller (us) first before involving the regulator. Once we have finished the study, we will keep some of the data so we can check the results. We will write our reports in a way that no-one can work out that you took part in the study.

For peer review only



CARDIFF UNIVERSITY PRIFYSGOL CAERDYDD

For peer review only - http://bmjopen.bmj.com/site/about/guidelines.xhtml

BMJ Open

# Pregnancy and Neonatal Outcomes in COVID-19: Study protocol for a global registry of women with suspected or confirmed SARS-CoV-2 infection in pregnancy and their neonates, understanding natural history to guide treatment and prevention

| | |
|---|---|
| Journal: | *BMJ Open* |
| Manuscript ID | bmjopen-2020-041247.R1 |
| Article Type: | Protocol |
| Date Submitted by the Author: | 26-Aug-2020 |
| Complete List of Authors: | Banerjee, Jayanta; Imperial College Healthcare NHS Trust, Department of Neonatology; Imperial College London Faculty of Medicine, Metabolism, Digestion and Reproduction<br>Mullins, E; Imperial College London, metabolism, digestion and reproduction; Imperial College<br>Townson, Julia; Cardiff University, Centre for Trials Research<br>Playle, Rebecca; Cardiff University, Centre for Trials Research<br>Shaw, Caroline; Imperial College London, Institute for reproductive and developmental biology<br>Kirby, Nigel; Cardiff University, Centre for Trials Research<br>Munnery, Kim; Cardiff University, Centre for Trials Research<br>Bourne, Tom; Imperial College London, Obstetrics and Gynaecology<br>Teoh, Tg; Imperial College Healthcare NHS Trust, Obstetrics and Gynaecology<br>Dhanjal, Mandish; Imperial College Healthcare NHS Trust, Obstetrics and Gynaecology<br>Poon, Liona; The Chinese University of Hong Kong, Obstetrics and Gynaecology<br>Wright, Alison; Royal Free London NHS Foundation Trust, Obstetrics and Gynaecology<br>Lees, Christoph; Imperial College London, Obstetrics and Gynaecology, Queen Charlotte's and Chelsea Hospital |
| <b>Primary Subject Heading</b>: | Infectious diseases |
| Secondary Subject Heading: | Obstetrics and gynaecology, Paediatrics, Public health |
| Keywords: | PUBLIC HEALTH, OBSTETRICS, NEONATOLOGY, INFECTIOUS DISEASES |
| | |

SCHOLARONE™
Manuscripts

*I, the Submitting Author has the right to grant and does grant on behalf of all authors of the Work (as defined in the below author licence), an exclusive licence and/or a non-exclusive licence for contributions from authors who are: i) UK Crown employees; ii) where BMJ has agreed a CC-BY licence shall apply, and/or iii) in accordance with the terms applicable for US Federal Government officers or employees acting as part of their official duties; on a worldwide, perpetual, irrevocable, royalty-free basis to BMJ Publishing Group Ltd ("BMJ") its licensees and where the relevant Journal is co-owned by BMJ to the co-owners of the Journal, to publish the Work in this journal and any other BMJ products and to exploit all rights, as set out in our licence.*

*The Submitting Author accepts and understands that any supply made under these terms is made by BMJ to the Submitting Author unless you are acting as an employee on behalf of your employer or a postgraduate student of an affiliated institution which is paying any applicable article publishing charge ("APC") for Open Access articles. Where the Submitting Author wishes to make the Work available on an Open Access basis (and intends to pay the relevant APC), the terms of reuse of such Open Access shall be governed by a Creative Commons licence – details of these licences and which Creative Commons licence will apply to this Work are set out in our licence referred to above.*

*Other than as permitted in any relevant BMJ Author's Self Archiving Policies, I confirm this Work has not been accepted for publication elsewhere, is not being considered for publication elsewhere and does not duplicate material already published. I confirm all authors consent to publication of this Work and authorise the granting of this licence.*

For peer review only

# Pregnancy and Neonatal Outcomes in COVID-19: Study protocol for a global registry of women with suspected or confirmed SARS-CoV-2 infection in pregnancy and their neonates, understanding natural history to guide treatment and prevention

Short title: Pregnancy and Neonatal outcomes in COVID-19 international registry

Author list

Joint first author
Dr Jayanta Banerjee, MD (Res)
Neonatal consultant and Hon Clinical Sen Lecturer
Du Cane Road, London, W12 0HS
Imperial College Healthcare NHS Trust
Institute of Reproductive and Developmental Biology, Imperial College London
jayanta.banerjee@ nhs.net
+44 77 7182 6045

Joint first author
Dr Edward Mullins, PhD
Clinical Lecturer in Obstetrics and Gynaecology
Du Cane Road, London, W12 0HS
Department of Metabolism, Digestion and Reproduction, Imperial College London
Edward.mullins@ imperial.ac.uk
+44 78 8102 9947

Julia Townson, BA
Research Fellow/Senior Trial Manager,
Centre for Trials Research, College of Biomedical and Life Sciences
Cardiff University
townson@ cardiff.ac.uk
+44 29 2068 7606

Dr Rebecca Playle, PhD
Deputy Director of Statistics
Centre for Trials Research, College of Biomedical and Life Sciences
Cardiff University
playlera@ cardiff.ac.uk
+44 29 2068 7516

Dr Caroline Shaw
Clinical Lecturer in Obstetrics
Du Cane Road, London, W12 0HS
Institute of Reproductive and Developmental Biology, Imperial College London
c.shaw13@ imperial.ac.uk

Nigel Kirby
Senior Data Manager
Centre for Trials Research, College of Biomedical and Life Sciences
Cardiff University

For peer review only

KirbyN@cardiff.ac.uk

Kim Munnery
Data Manager
Centre for Trials Research, College of Biomedical and Life Sciences
Cardiff University
MunneryK1@cardiff.ac.uk

Prof Tom Bourne, PhD
Professor of Practice, Imperial College London
Consultant Gyanecologist, Queen Charlotte's and Chelsea Hospital
tbourne@imperial.ac.uk
+44 77 6807 6797

Professor Tg Teoh,
Professor and Consultant Obstetrician
St Marys Hospital
Imperial College Healthcare NHS Trust
Tg.teoh@ nhs.net

Dr Mandish Dhanjal, BSc
Consultant Obstetrician and Gynaecologist
Queen Charlotte's and Chelsea Hospital
Imperial College Healthcare NHS Trust
Mandish.Dhanjal@ nhs.net
+44 7958 323066

Prof Liona C. Poon
Professor Department of Obstetrics and Gynaecology
The Chinese University of Hong Kong
liona.poon@ cuhk.edu.hk
+852 61089496

Dr Alison Wright
Consultant Obstetrician and Gynaecologist
Royal Free Hospital
Alison.Wright15@ nhs.net
+44 7702269002

Corresponding author Prof Christoph Lees, MD
Professor of Obstetrics and Hon Consultant Obstetrician
Lead, Centre for Fetal Care
Imperial College Healthcare NHS Trust
Institute of Reproductive and Developmental Biology, Imperial College London
c.lees@imperial.ac.uk
+44 77 8526 8351

For peer review only

# Abstract

## Introduction

Previous novel coronavirus pandemics, Severe Acute Respiratory Syndrome (SARS) and Middle East Respiratory Syndrome (MERS), observed an association of infection in pregnancy with pre-term delivery, stillbirth and increased maternal mortality. Coronavirus disease 2019 (COVID-19), caused by SARS-CoV-2 infection, is the largest pandemic in living memory.

Rapid accrual of robust case data on women in pregnancy and their babies affected by suspected COVID-19 or confirmed SARS-CoV-2 infection will inform clinical management and preventative strategies in the current pandemic and future outbreaks.

## Methods and analysis

The Pregnancy And Neonatal outcomes in COVID-19 (PAN-COVID) registry is an observational study collecting focussed data on outcomes of pregnant mothers who have had suspected COVID-19 in pregnancy or confirmed SARS-CoV-2 infection and their neonates via a web-portal. Amongst the women recruited to the PAN-COVID registry, the study will evaluate the incidence of:

1. Miscarriage and pregnancy loss
2. FGR and stillbirth
3. Pre-term delivery
4. Vertical transmission (suspected or confirmed) and early-onset neonatal SARS-CoV-2 infection

Data will be centre based and collected on individual women and their babies. Verbal consent will be obtained, to reduce face-to-face contact in the pandemic whilst allowing identifiable data collection for linkage. Statistical analysis of the data will be carried out on a pseudonymised dataset by the study statistician. Regular reports will be distributed to collaborators on the study research questions.

## Ethics and dissemination

This study has received research ethics approval in the UK. For international centres, evidence of appropriate local approval will be required to participate, prior to entry of data to the database. The reports will be published regularly. The outputs of the study will be regularly disseminated to


For peer review only

participants and collaborators on the study website (https://pan-covid.org) and social media channels as well as dissemination to scientific meetings and journals.

## Study Registration number

The study is registered with ISRCTN68026880

For peer review only

Article summary

## Strengths and limitations of the study

- The PAN-COVID registry aims to collect an international dataset, which will enable us to answer a focussed set of questions related to pregnancy, maternal and neonatal outcomes of SARS-CoV-2 infection in pregnancy.

- There is a high likelihood of an adequate sample size to allow us to measure all the stated focussed outcomes of SARS-CoV-2 infection in pregnancy.

- The PAN-COVID protocol facilitates data sharing and collaboration with multiple global partners.

- This registry establishes a collaborative framework, which can be used to respond effectively to guide care for women in pregnancy and their neonates in future pandemics.

- Registry studies are generally unlikely to fully capture all cases and therefore carry risk of underestimating infection rates and over-estimating infection complication and fatality rates.

For peer review only


For peer review only - http://bmjopen.bmj.com/site/about/guidelines.xhtml

# Introduction

The majority of all new, emerging, or re-emerging viruses affecting the human population in the recent years are zoonoses, including HIV/AIDS, severe acute respiratory syndrome (SARS), the H5N1 strain of avian influenza, the 2009 pandemic H1N1 influenza virus, the Middle East respiratory syndrome (MERS), the Zika and Nipa viruses[1][2] and most recently Coronavirus disease 2019 (COVID-19) caused by SARS-CoV-2 virus. The spread of these infections in an immune-naïve human population, as shown by the recent COVID-19 pandemic poses serious strain on public health. To tackle such large pandemics, international collaborative and multidisciplinary approaches are necessary.

These should aim to identify those at highest risk of exposure to emerging pathogens, to characterise the culture and practices that modify their risk (e.g. the presence of live animal markets and the use of face masks in South-East Asia) and to develop preventative interventions accordingly [3][4]. Pregnant women infected with SARS or MERS were at increased risk of mortality and morbidities such as stillbirth, preterm birth and fetal growth restriction (FGR)[5][6]. Case series for pregnant women infected during outbreaks of SARS [7] or MERS[8] report a variety of different outcomes, with variable recording of diagnostic testing, maternal, fetal and neonatal outcomes, and the presence or absence of vertical transmission. Clinical outcomes appear worse for pregnant compared with non-pregnant women infected with SARS and H1N1 influenza[7][9].

As of the 12$^{th}$ May 2020, after disambiguation and removal of duplicate reporting, the rolling review of all cases worldwide conducted by Thornton et al.[10] contains 92 publications including details of 806 pregnant women and 674 fetuses. At the time of writing this protocol, 457 of the included women had delivered and were alive and 21 women had died. These women had delivered 428 babies, of which 94 were born pre-term, 17 were infected with COVID-19, and there were 15 neonatal deaths. The largest study to date has been from the UK (Knight et al) who reported 427 cases (247 competed pregnancies): there were 5 (1%) maternal deaths, 3 (1%) stillbirths, and 2 (1%) neonatal deaths[11]. There are currently limited data on the effect of COVID-19 on second trimester miscarriage [12] and no available data on first trimester miscarriage or growth restriction. The risk of reported probable vertical transmission of SARS-CoV-2 infection is estimated at 2.5% in women admitted to hospital with COVID-19; with case reports describing increased SARS-CoV-2 IgM levels in neonates, and pneumonia and



For peer review only - http://bmjopen.bmj.com/site/about/guidelines.xhtml

lymphopaenia in neonates with negative COVID-19 RT-PCR testing, principally from naso-pharyngeal swabs [13][14].

There are major knowledge gaps on the effect of COVID-19 on various stages of pregnancy, and its effect on fetuses in terms of growth restriction, prematurity and short and long term morbidities[15]. There is an urgent need to collect case data rapidly, to pool global data on the natural history of women affected by suspected COVID-19 or confirmed SARS-CoV-2 in pregnancy to inform treatment and implement preventative strategies in the current and future outbreaks. Published case series are almost always out of date when published and cases may overlap. A centre-based registry, gathering case data prospectively on the effect of SARS-CoV-2 infection from healthcare systems around the world offers a method to accrue clinical outcomes on key research questions from a variety of populations and healthcare systems without these limitations.

PAN-COVID registry will focus on miscarriage, fetal growth restriction, stillbirth, pre-term delivery and vertical transmission (suspected or confirmed) and early-onset neonatal SARS-CoV-2 infection and will include fields on ultrasound diagnosis and neonatal care not included in other more general studies. This study will identify cases which should also be reported to International Network of Obstetric Survey Systems (INOSS) population-based surveillance systems of confirmed COVID-19 cases admitted to hospital through existing reporting mechanisms. The UK's obstetric surveillance system (UKOSS) is a population surveillance study, reporting women in pregnancy hospitalized with COVID-19 or SARS-CoV-2 infection. The PAN-COVID registry is distinct in that it will collect, via a web portal, data on SARS-CoV-2 infections in pregnancy, whether the diagnosis is presumptive based on symptoms or following a positive test and whether the patient is admitted to hospital or not. Given that the SARS-CoV-2 virus is likely to affect a high proportion of the global population and adverse outcomes may influence policy and practice in a short time-frame, 'real-time' high level reporting on a regular basis will be valuable for UK and international clinicians and policy makers.

PAN-COVID registry will work collaboratively with approved studies and registries to ensure that the global impact of the pandemic is captured as fully as possible, with data sharing and linkage. PAN-COVID has established links with the UK National Neonatal Research Database (NNRD) (REC Reference: 16/LO/1093) who will provide data on neonatal outcomes, and the British Paediatric Surveillance Unit (BPSU) with whom there is in principle an agreement for data sharing.

For peer review only



For peer review only - http://bmjopen.bmj.com/site/about/guidelines.xhtml

In the UK, the PAN-COVID registry will signpost to UKOSS where the case definition requires a more detailed data collection. We are in discussions with several international studies to agree on a common dataset to enable future merging of information.

## Methods and analysis

This is an observational global pregnancy and neonatal register (PAN-COVID registry) collecting outcome data from women in pregnancy who have confirmed SARS-CoV-2 infection or signs and symptoms of COVID-19 during their pregnancy. The study is sponsored by Imperial College London and funded by UK Research Institute (UKRI) and NIHR.

The main study objectives are, firstly, to establish an UK and international disease registry for women with suspected COVID-19 or confirmed SARS-CoV-2 infection in pregnancy; in the UK this will be linked to neonatal data (from NNRD). Secondly, PAN-COVID investigators aim to publish regular reports focused on our principle research question: in women recruited to the PAN-COVID registry with 1) Suspected COVID-19 or 2) Confirmed SARS-CoV-2 infection, what is the incidence of (a) miscarriage, (b) fetal growth restriction and stillbirth, (c) pre-term birth and (d) Suspected or confirmed vertical transmission to the neonate and perinatal infection.

### Data entry

*Demographics* – date of birth, NHS number (or international equivalent), smoking status, last menstrual period, expected date of delivery, body mass index (BMI), history of previous pregnancies/miscarriages/loss/small for gestational age/delivery (pre-term/term), co-morbidities, medications, ethnicity, number of fetuses, structural abnormality on ultrasound.

*COVID-19 signs and symptoms* – fever, new persistent cough, anosmia, myalgia, diarrhoea, shortness of breath, fatigue, abdominal pain, chest pain, hoarseness of voice, loss of appetite, delirium.

*SARS-CoV-2 infection* – SARS CoV 2 test performed, date of test(s), results of test(s) (if available)

*COVID-19 treatment* – requirement for (including duration of) inpatient, level 2 or 3 critical care, supplementary oxygen, non-invasive ventilation, endo-tracheal intubation, extracorporeal membrane oxygenation, antiviral/other specific anti-COVID-19 therapy, iatrogenic delivery or termination due to maternal compromised from COVID-19.

*Maternal outcomes* – non-invasive ventilation, intubation and ventilation and maternal death.



For peer review only - http://bmjopen.bmj.com/site/about/guidelines.xhtml

*Co-morbidities* – Hypertension, respiratory disease, cardiovascular disease, renal disease, autoimmune disease, medications including aspirin, progesterone, others.

*Delivery details* – iatrogenic/spontaneous, date of delivery/miscarriage, mode of delivery, livebirth/stillbirth/miscarriage, birthweight, sex.

*Postnatal outcomes* – breastfeeding, baby cared for separately from mother until discharge

*Neonatal outcomes* – date of birth, birthweight, gestation at birth, APGAR at 5 minutes, neonatal death, COVID-19 transmission (neonate with positive swab/serology), congenital abnormality/deformation, date of discharge from hospital, re-admission up to 28 days of life.

## Study population

Study Centres will be asked to identify eligible women of childbearing age (18-50 years) at the time of an early pregnancy attendance or at delivery who have had suspected COVID-19 or confirmed SARS-CoV-2 infection in pregnancy. Case data will be entered into the online registry. The study will recruit women affected by suspected COVID-19 or confirmed SARS-CoV-2 infection from January 2020 and March 2021. Participants will be able to withdraw from the register at any time without affecting their medical care. Participants should inform any member of the study team of their wish to withdraw consent. When a participant withdraws consent, they should be asked whether they are willing to allow ongoing data collection, such as maternal and fetal outcomes, from their medical records. If the participant withdraws consent and does not wish to have any further data collected, then this will be recorded on the database and no further data entry will be possible.

## Outcomes

Assessment of outcomes will require follow up by individual healthcare professionals, by accessing medical records routinely available to them as part of the clinical care team. When a pregnant woman with SARs-CoV-2 infection or suspected COVID 19 is registered on the database, they will automatically be assigned a unique participant identification number. Date of birth and NHS number, or equivalent in non-UK centres, will be stored on the database. The limit for data collection will be 28 days after the delivery or pregnancy loss of the last woman registered. Linkage will allow data up to 2 years of age to be collected for pre-term babies in the UK with the NNRD database.

## Data collection pathway

For peer review only

The Centre for Trials Research (CTR), Cardiff University, has designed a registry and web portal for data entry for healthcare professionals. The CTR hosts and maintans the study web page (https://pan-covid.org) which allows maternity healthcare providers to register interest in submitting data to an online database. The data manager will manage the registration of healthcare professionals, keeping personal identifiable information on a separate, password protected, spreadsheet with restricted access. The study co-ordinator will manage registration of healthcare professionals and work with the data manager to provide login permission to those who wish to contribute to the register. Login permission will only be granted to healthcare professionals who have confirmed their email address, which must be from a bona fide healthcare or educational organisation and local agreement and ethical approval is in place.

Where a web portal cannot be accessed by the investigators, a bespoke spreadsheet has been designed to allow automatic uploading of data to the main registry database.

## Analysis

Statistical analysis of the data will be carried out a pseudonymised dataset containing NHS number (or equivalent international data field). Numerical data for cases reported and outcomes (miscarriage, FGR, stillbirth, pre-term delivery and vertical transmission) will be reported in anonymised, aggregate regular reports, published on the study website.

## Sample size

Pre-specified sample size estimation was not carried out given the aim of this observational study was to collate all consecutive eligible case outcomes in participating centres between data collection start and 18 months from start of data collection. It may be useful to provide an approximate guide as to the width of the 95% confidence interval that can be achieved for proportions using historical data from the UK. These historical data may not hold during the current pandemic and may alter due to multiple reasons such as access to health care or behaviour changes. Contemporaneous data collected using other current national registries will provide true background rates for comparative purposes at the end of the study period. Generalising these data internationally may not be possible unless similar contemporaneous data exist for comparison.

The estimated representative pre-COVID pandemic incidences of miscarriage, SGA and stillbirth in the UK were 30%, 10%, 0.2% respectively. The expected outcome proportions during the


For peer review only - http://bmjopen.bmj.com/site/about/guidelines.xhtml

COVID pandemic are 40%, 15% and 0.4% respectively. A sample size of 500 would allow the estimation of the width of 95% confidence intervals for the proportion of miscarriage as 40% +/- 4.2%, for SGA 15% +/- 2.9% and for still birth 0.4% +/-0.3%.

These figures are meant only as a guide and reference proportions may change over this period. Since attaining sufficient precision for stillbirth requires by far the largest sample size then study recruitment targets could be based on this rarest of outcomes. This would then provide sufficient precision to assess other more common outcomes. These figures are based on data from the UK only. Reference proportions vary by country but are of a similar order to those in the UK.

Appropriate quantitative analyses will be conducted by a study statistician. Dates collected from sites for expected data of delivery or last menstrual period, week of pregnancy at delivery and date of any COVID tests in the participant or neonate will be cross-checked and validated for the final report. Regular interim reports will use contemporaneous data as it is collated.

Derived variables to be reported are:

- Infection delivery interval (<2 or ≥ 2 weeks after onset of suspected or confirmed COVID-19
- Week of pregnancy when COVID-19 diagnosis was made
- Number of days from delivery to SARS-COV-2 test
- Birth weight centiles adjusted for gender and gestational age

Fortnightly bulletins will consist of; counts of participants by country, proportions for maternal COVID-19 diagnosis status (suspected, positive test, negative test), week of pregnancy for COVID-19 diagnosis (<23 weeks, 23-36 weeks, ≥37 weeks), weeks of pregnancy at delivery (<23 weeks, 23-36 weeks, ≥37 weeks), maternal and neonatal mortality, outcome of delivery (live birth, miscarriage, intrauterine death, still birth (>22+6 weeks gestation)), fetal growth restriction split by delivery interval (<2 vs ≥2 weeks), COVID-19 diagnosis status for babies split by number of days from delivery to test (0, 1-7, 8-28) and birth weight centiles. Mean (SD) and median birth weights will also be reported. Further analysis will take place at one interim time point and at final data collection. For the interim analysis a subset of validated outcomes will be reported consisting of total cohort proportions of; gestation at delivery, birthweight z-scores, perinatal mortality (still birth, early neonatal death), neonatal COVID-19 or SARS-COV-2 swab positive, neonatal morbidity and maternal death. These data will be split by suspected and confirmed COVID-19

For peer review only

diagnosis. Additional graphical exploratory analysis of the data will assess any possible association between the infection delivery interval and the timing of delivery as well as birthweight. The final analysis will report all demographic baseline data for the whole cohort and all outcomes as well as these graphical trends. Proportional data for the study outcomes will be presented with 95% confidence intervals, by country, where numbers allow. Data and all appropriate documentation will be stored for a minimum of 10 years after the completion of the study, including the follow-up period.

## Ethics and dissemination

The Study Coordination Centre has obtained approval from the Haydock Research Ethics Committee (REC) and Health Regulator Authority (HRA), REC reference: 20/NW/0212 and had been considered to be an Urgent Public Health (UPH) study by the UK Clinical Research Network (UK CRN).

The study must also receive confirmation of capacity and capability from each participating NHS Trusts in the UK before accepting participants into the study or any research activity is carried out. The study will be conducted in accordance with the recommendations for physicians involved in research on human subjects adopted by the 18th World Medical Assembly, Helsinki 1964 and later revisions. The international participating centres will be required to obtain appropriate local approval from their centres. All those taking consent will be qualified and trained to take consent in a medical context. To reduce face to face consultations during the pandemic verbal consent will be obtained after provision of the participant information sheet: PIS (Appendix 1) which will have details of how to contact the PAN-COVID study team, if they would like further information or to withdraw from the study and this will be recorded in the patients notes and on the study database. Where women have lost capacity to provide consent and have confirmed COVID-19, women will be assumed to provide consent. If they regained capacity, the study will be explained to them and they will be provided with the PIS , which will have details of how to contact the PAN-COVID study team, if they would like further information or to withdraw from the study. If they choose to withdraw, data pertaining to their care would be removed from the registry. Where feasible, the woman's partner or a family member will be provided with the PIS and asked if they feel able to advise on the presumed wishes and feelings of participants unable to consent for themselves and on their inclusion in the research. Their response will be recorded on the database and in the



For peer review only - http://bmjopen.bmj.com/site/about/guidelines.xhtml

participants clinical notes. These models will give a reasonable opportunity for women to provide their data to the study, whilst being mindful not to over burden healthcare resources. Research has shown that within a pandemic, potential participants and their families are broadly agreeable to alternative methods of gaining consent, therefore we decided upon this verbal model of consent, as a pragmatic solution to ensure women are informed of how their data will be used, and alleviate additional burden to healthcare resources [16]. The Chief Investigators (CCL and EM) will preserve the confidentiality of participants taking part in the study and is registered under the General Data Protection Regulation (GDPR) 2018. In terms of indemnity, Imperial College London holds negligent harm and non-negligent harm insurance policies which apply to this study. Imperial College London will act as the Sponsor for this study. Delegated responsibilities will be assigned to the NHS trusts taking part in this study. The study may be subject to inspection and audit by Imperial College London under their remit as sponsor and other regulatory bodies to ensure adherence to GCP and the UK Policy Framework for Health and Social Care Research.

## Data security

Cardiff University has strict policies and processes in place to ensure adherence to the General Data Protection Regulation 2018. The University is registered on the Information Commissioner's Office Data Protection register, number Z6549747. All data collected on the Registry will be held on Cardiff University servers, which have a high level of security. CTR database developers have run a security check on the trials.cardiff.ac.uk server and it has passed to an acceptable standard to EU and non-EU centres.

The person allocating Centre IDs and logins will not have access to the database fields containing personal information. For healthcare professionals to enter follow up data they will need to maintain a secure spreadsheet (password protected) with participants' identifiable information logged against their unique participant identification number.

To reduce any potential risk of identification of study participants, access will be restricted to the section of the database containing identifiable information (NHS number, date of birth, ethnicity) to those responsible for maintaining the database (two individuals). This will mean that the CTR members of the research team (two different individuals) responsible for allocating the unique centre and participant IDs will not be able access any identifiable data. In addition, all other CTR members of the research team will be blinded to which centre ID relates to which centre. For statistical analyses, a dataset will be prepared by the database developers which will have all NHS


For peer review only - http://bmjopen.bmj.com/site/about/guidelines.xhtml

numbers, and the international equivalent, removed, reducing possible identification of participants further.

Patient and public involvement

Patients and public were not involved in the initial research idea and study design, due to the rapid response required at the start of the COVID 19 pandemic and lockdown. The NIHR funded this research and involves patients and public in setting research priorities and in reviewing funding applications. Patients and the public will be notified of the results through our established links within the UK including the premature baby charity Bliss and internationally through EFCNI in Europe and with our US partner the AAP. We expect that the major publications from this study will be widely cited in the scientific and lay press and this will prove to be the most effective route of dissemination.

## Dissemination

Regular reports of case data will be made available on the study website. Although members of the public were not involved in the study design as it is broadly a registry, the maternity and neonatal focus groups will be involved in the dissemination of the outputs. The outputs of the PAN-COVID registry will be presented in national and international conferences, lay and digital social media and published in peer-reviewed scientific journals which will be made available as open-access, as per the NIHR's publication policy.

For peer review only

# References


[1] D. Di Mascio *et al.*, "Outcome of Coronavirus spectrum infections (SARS, MERS, COVID 1 -19) during pregnancy: a systematic review and meta-analysis," *Am. J. Obstet. Gynecol. MFM*, 2020.

[2] M. Elisabeth Lopes Moreira *et al.*, "Neurodevelopment in infants exposed to Zika virus in utero," *New England Journal of Medicine*. 2018.

[3] K. J. Olival, P. R. Hosseini, C. Zambrana-Torrelio, N. Ross, T. L. Bogich, and P. Daszak, "Host and viral traits predict zoonotic spillover from mammals," *Nature*, 2017.

[4] D. Joly *et al.*, "The first phase of PREDICT: Surveillance for emerging infectious zoonotic diseases of wildlife origin (2009-2014)," *Int. J. Infect. Dis.*, 2016.

[5] A. M. Li and P. C. Ng, "Severe acute respiratory syndrome (SARS) in neonates and children," *Archives of Disease in Childhood: Fetal and Neonatal Edition*. 2005.

[6] C. C. Shek *et al.*, "Infants born to mothers with severe acute respiratory syndrome.," *Pediatrics*, 2003.

[7] S. F. Wong *et al.*, "Pregnancy and perinatal outcomes of women with severe acute respiratory syndrome," *Am. J. Obstet. Gynecol.*, 2004.

[8] S. H. Alfaraj, J. A. Al-Tawfiq, and Z. A. Memish, "Middle East Respiratory Syndrome Coronavirus (MERS-CoV) infection during pregnancy: Report of two cases & review of the literature," *Journal of Microbiology, Immunology and Infection*. 2019.

[9] M. Pierce, J. J. Kurinczuk, P. Spark, P. Brocklehurst, and M. Knight, "Perinatal outcomes after maternal 2009/H1N1 infection: National cohort study," *BMJ*, 2011.

[10] J. et al Thornton, "Ripe tomato.org." [Online]. Available: https://ripe-tomato.org/2020/03/31/covid-19-in-pregnancy-running-totals/.

[11] M. Knight et al., "Characteristics and outcomes of pregnant women hospitalised with confirmed SARS-CoV-2 infection in the UK: a national cohort study using the UK Obstetric Surveillance System (UKOSS) The UK Obstetric Surveillance System SARS-CoV-2 Infection in Pregnancy Co," 2020.

[12] D. Baud *et al.*, "Second-Trimester Miscarriage in a Pregnant Woman With SARS-CoV-2 Infection," *JAMA - J. Am. Med. Assoc.*, 2020.

[13] H. Zeng *et al.*, "Antibodies in Infants Born to Mothers with COVID-19 Pneumonia," *JAMA - Journal of the American Medical Association*. 2020.


For peer review only



For peer review only - http://bmjopen.bmj.com/site/about/guidelines.xhtml

[14] C. Fan *et al.*, “Perinatal Transmission of COVID-19 Associated SARS-CoV-2: Should We Worry?,” *Clin. Infect. Dis.*, 2020.

[15] H. Liu, L.-L. Wang, S.-J. Zhao, J. Kwak-Kim, G. Mor, and A.-H. Liao, “Why are pregnant women susceptible to viral infection: an immunological viewpoint?,” *J. Reprod. Immunol.*, 2020.

[16] N. H. Gobat *et al.*, “Key stakeholder perceptions about consent to participate in acute illness research: A rapid, systematic review to inform epi/pandemic research preparedness,” *Trials*, 2015.

For peer review only

## Supplementary files

Participant information leaflet v.5.1 19/5/20

## Authors' contributions

CL and EM conceived of the study and prepared the study design. JB, TB, CS and JT helped in preparing the study design. JB prepared the first draft. RP, NK and KM helped in the statistical design of the registry, preparation of the online platform for recruitment and writing the method section of the manuscript. TT, MD, LP and AW helped in development of the study design and preparation of the manuscript. All authors contributed to the manuscript and agreed the final draft. All authors agreed to be accountable for all aspects of the work in ensuring that questions related to the accuracy or integrity of any part of the work are appropriately investigated and resolved.

## Funding statement

The PAN-COVID registry is funded by the United Kingdom Research Institute (UKRI) and National Institute of Health and Research (NIHR) through COVID-19 Rapid Response Call 2, grant reference MC_PC 19066

## Competing interest statement

None declared

For peer review only

PAN-COVID **PREGNANCY AND NEONATAL OUTCOMES IN COVID-19** Imperial College London

Pregnancy and Neonatal Outcomes in COVID-19: A global registry of women affected by COVID-19 in pregnancy and their neonates, understanding natural history to guide treatment and prevention

## Invitation to participate in early pregnancy

We are inviting women who have had suspected COVID-19 or confirmed SARS-CoV-2 infection (the virus that causes COVID-19) in their pregnancy to consent to join this research study, collecting information about their pregnancy. This form gives information about the study including the aims, risks and benefits of taking part.

**WHAT YOU SHOULD KNOW ABOUT THIS RESEARCH STUDY:**

**What is the purpose of the study?** This study aims to collect information about COVID-19 and SARS-CoV-2 in pregnancy from around the world into a register which the research team will use to share information with healthcare professionals around the world, allowing them to improve the care they give. We would like to find out more about the effect of COVID-19 on early pregnancy

Information will be held on a secure database and used anonymously to produce regular updates for healthcare professionals. The website with these reports and information is open to the public https://pan-covid.org/

**Why have I been chosen?** You have been chosen to consider taking part because you have had likely or confirmed COVID-19 during your pregnancy or just afterwards.

**Do I have to take part?** It is up to you to decide whether or not to take part. If you do decide to take part you will be given this information sheet to keep. If you decide to take part you are still free to withdraw at any time and without giving a reason. A decision to withdraw at any time, or a decision not to take part, will not affect the standard of care you receive. If you are happy to take part, we will ask for you to verbally give your consent, which will be recorded.

**What will happen to me if I take part?** Your healthcare professional will enter information about your pregnancy, the postnatal period and your baby into the secure study website. **This will include your NHS number, your date of birth, the key dates in the pregnancy and information about your health and the outcomes of the pregnancy. You will not be contacted again. We will use the NHS numbers to link the data collected to your routinely available health information**

**What are the possible benefits of taking part?** There will be no direct benefit to you from taking part in this study. We aim to use the information collected for this register to improve the understanding of COVID-19 in pregnancy and help healthcare professionals to improve treatment and prevention of the disease.

**What are the possible risks of taking part?** There are no risks that we can foresee from taking part in this study as we will only be collecting data.

**What will happen to the results of the research study?** Results of this study will be published on the study website, in medical journals and presented at medical conferences. You will not be identified in any report or publication.

**Who is organising and funding the research?** This study is funded by the UK Medical Research Council

**Who has reviewed the study?** This study was given a favourable ethical opinion for conduct in the NHS by Haydock Research Ethics Committee.

**What if something goes wrong?** If you are harmed by taking part in this research project, there are no special compensation arrangements. If you are harmed due to someone's negligence, then you may have grounds for a legal action. Regardless of this, if you wish to complain, or have any concerns about any aspect of the way you have been treated during the course of this study then you should immediately inform the Investigator Christoph Lees, c.lees@imperial.ac.uk . The normal National Health Service complaints mechanisms are also available to you



CARDIFF UNIVERSITY PRIFYSGOL CAERDYDD

For peer review only - http://bmjopen.bmj.com/site/about/guidelines.xhtml

PAN-COVID

# PREGNANCY AND NEONATAL OUTCOMES IN COVID-19

Imperial College London

Pregnancy and Neonatal Outcomes in COVID-19: A global registry of women affected by COVID-19 in pregnancy and their neonates, understanding natural history to guide treatment and prevention

**How will we use information about you and your baby?** Imperial College London is the sponsor for this study and will act as the data controller for this study. This means that we are responsible for looking after your information and using it properly. Imperial College London will keep your personal data for 10 years after the study has completed in relation to primary research data. We will need to use information from your medical records for this research project.

This information will include information about your pregnancy and general health. People will use this information to do the research or to check your records to make sure that the research is being done properly. We will keep all information about you safe and secure.

**Legal basis** As a university we use personally-identifiable information to conduct research to improve health, care and services. As a publicly-funded organisation, we have to ensure that it is in the public interest when we use personally-identifiable information from people who have agreed to take part in research. This means that when you agree to take part in a research study, we will use your data in the ways needed to conduct and analyse the research study. Health and care research should serve the public interest, which means that we have to demonstrate that our research serves the interests of society as a whole. We do this by following the UK Policy Framework for Health and Social Care Research.

**International transfers** There may be a requirement to transfer information to countries outside the European Economic Area (for example, to a research partner). Where this information contains your personal data, Imperial College London will ensure that it is transferred in accordance with data protection legislation. If the data is transferred to a country which is not subject to a European Commission (**EC**) adequacy decision in respect of its data protection standards, Imperial College London will enter into a data sharing agreement with the recipient organisation that incorporates EC approved standard contractual clauses that safeguard how your personal data is processed.

**Sharing your information with others** For the purposes referred to in this privacy notice and relying on the bases for processing as set out above, we will share your personal data with certain third parties.

- Other Imperial College employees, agents, contractors and service providers (for example, suppliers of printing and mailing services, email communication services or web services, or suppliers who help us carry out any of the activities described above). Our third party service providers are required to enter into data processing agreements with us. We only permit them to process your personal data for specified purposes and in accordance with our policies.
- Cardiff University, who are running the database for the study

**What are your choices about how your information is used?** You can stop being part of the study at any time, without giving a reason, by emailing c.lees@imperial.ac.uk but we will keep information about you that we already have.

- If you choose to stop taking part in the study, we would like to continue collecting information about your health from your hospital. If you do not want this to happen, tell us and we will stop.
- We need to manage your records in specific ways for the research to be reliable. This means that we won't be able to let you see or change the data we hold about you.

**Where can you find out more about how your information is used** You can find out more about how we use your information

- at www.hra.nhs.uk/information-about-patients/
- by asking one of the research team
- by sending an email to pan-covid@cardiff.ac.uk

**Complaint** If you wish to raise a complaint on how we have handled your personal data, please contact Imperial College London's Data Protection Officer via email at dpo@imperial.ac.uk, via telephone on 020 7594 3502 and/or via post at Imperial College London, Data Protection Officer, Faculty Building Level 4, London SW7 2AZ. If you are not satisfied with our response or believe we are processing your personal data in a way that is not lawful you can complain to the Information Commissioner's Office (ICO). The ICO does recommend that you seek to resolve matters with the data controller (us) first before involving the regulator. Once we have finished the study, we will keep some of the data so we can check the results. We will write our reports in a way that no-one can work out that you took part in the study.



Cardiff University Prifysgol Caerdydd

For peer review only - http://bmjopen.bmj.com/site/about/guidelines.xhtml
